# Assimilating Satellite Land Surface States Data from Fengyun-4A

**DOI:** 10.1038/s41598-019-55733-3

**Published:** 2019-12-20

**Authors:** Chunlei Meng, Huoqing Li

**Affiliations:** 1 Institute of Urban Meteorology, China Meteorological Administration, 100089 Beijing, China; 2 Institute of Desert Meteorology, China Meteorological Administration, 830002 Urumqi, Xinjiang China

**Keywords:** Atmospheric science, Atmospheric dynamics

## Abstract

Fengyun-4A is the new generation of Chinese geostationary meteorological satellites. Land surface albedo, land surface emissivity and land surface temperature are key states for land surface modelling. In this paper, the land surface albedo, land surface emissivity and land surface temperature data from Fengyun-4A were assimilated into the Integrated Urban land Model. The Fengyun-4A data are one of the data sources for the land data assimilation system which devoted to produce the high spatial and temporal resolution, multiple parameters near real-time land data sets. The Moderate-Resolution Imaging Spectroradiometer (MODIS) LSA and LSE data, the Institute of Atmospheric Physics, China Academy of Sciences (IAP) 325 m tower observation data and the observed 5 cm and 10 cm soil temperature data in more than 100 sites are used for validation. The results indicate the MODIS land surface albedo is much smaller than the Fengyun-4A and is superior to the Fengyun-4A for the Institute of Atmospheric Physics, China Academy of Sciences 325 m tower site. The Moderate-Resolution Imaging Spectroradiometer land surface emissivity is smaller than the Fengyun-4A in barren land surface and the differences is relatively small for other land use and land cover categories. In most regions of the research area, the Fengyun-4A land surface albedo and land surface emissivity are larger than those of the simulations. After the land surface albedo assimilation, in most regions the simulated net radiation was decreased. After the land surface emissivity assimilation, in most regions the simulated net radiation was increased. After the land surface temperature assimilation, the biases of the land surface temperature were decreased apparently; the biases of the daily average 5 cm and 10 cm soil temperature were decreased.

## Introduction

Fengyun-4A is the new generation of Chinese geostationary meteorological satellites with greatly enhanced capabilities for high-impact weather event monitoring, warning, and forecasting^1^. For land surface models, land surface albedo (LSA), land surface emissivity (LSE) and land surface temperature (LST) are important states which could be retrieved from Fengyun-4A satellite. In order to improve the performance of the land surface model and make use of the Fengyun-4A data sufficiently, these land surface states retrieved from Fengyun-4A should be validated and assimilated to land surface models.

LSA is defined as the surface reflected solar radiation divided by the surface incident solar radiation^2^. It is associated with the land surface categories^3^, soil moisture^4^, solar zenith angle^5^ and the weather conditions^6^. LSA determines the amount of the upward shortwave radiation, so it is an important state in surface energy balance and shapes the earth's climate and climate change^7–11^. The Moderate-Resolution Imaging Spectroradiometer (MODIS) LSA product is one of the widely used and validated^12–20^ products; and it is also assimilated into the land surface models^21,22^.

LSE^23^ and LST determine the amount of the upward longwave radiation. LSE is important to obtain the LST^24,25^. LSE could be estimated by using physical modeling method^26^; the MODIS LSE data^27,28^ are also used widely and assimilated into the land surface models. The LST and soil moisture are the prognostic variables of the surface energy and water balance model. They are assimilated separately or simultaneously using different assimilation methods^29–34^.

As a new launching satellite, Fengyun-4A data was not validated and assimilated. In this paper, the LSA, LSE and LST data from Fengyun-4A were assimilated into the Integrated Urban land Model (IUM)^35^. The Fengyun-4A data are one of the data sources for the land data assimilation system which devoted to produce the high spatial and temporal resolution, multiple parameters near real-time land data sets. The MODIS LSA and LSE data, the Institute of Atmospheric Physics, China Academy of Sciences (IAP) 325 m tower observation data and the observed 5 cm and 10 cm soil temperature data in more than 100 sites are used for validation. The four-dimensional variational assimilation method^36,37^ was used to assimilate the Fengyun-4A LST data. As the evaporative fractions (EFs) do not vary much especially in the clear day^38^, they were used as the tuned factors to simplify the variational assimilation approach.

Section 2 is the data used in this paper. Section 3 describes the model and the assimilation method. Section 4 demonstrates the results and discussion of the LSA, LSE and LST assimilation. The conclusions of this study are discussed in the last section.

## Data

### Case study

In general, eastern China is a geographical and loosely defined region that covers the eastern part of China. In this study, eastern China is located at 20°–45°N, 105°–125°E, where the population density and urbanization degree are relatively high.

### Data

#### Data used for the model

**Figure 1:**
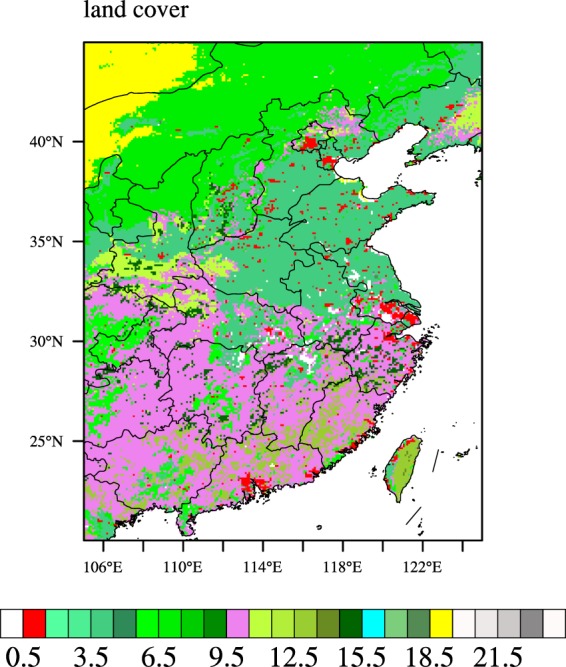
MODIS LULC in eastern China (0. Ocean. 1 Urban and Built-Up Land. 2 Dryland Cropland and Pasture. 3 Irrigated Cropland and Pasture. 4 Mixed Dryland/Irrigated Cropland and Pasture. 5 Cropland/Grassland Mosaic. 6 Cropland/Woodland Mosaic. 7 Grassland. 8 Shrubland. 9 Mixed Shrubland/Grassland. 10 Savanna. 11 Deciduous Broadleaf Forest. 12 Deciduous Needleleaf Forest. 13 Evergreen Broadleaf Forest. 14 Evergreen Needleleaf Forest. 15 Mixed Forest. 16 Water Bodies. 17 Herbaceous Wetland. 18 Wooded Wetland. 19 Barren or Sparsely Vegetated. 20 Herbaceous Tundra. 21 Wooded Tundra. 22 Mixed Tundra. 23 Bare Ground Tundra. 24 Snow or Ice). (Generated by NCAR Command Language (NCL) Version 6.3.0, ref. ^44^).

**Figure 2:**
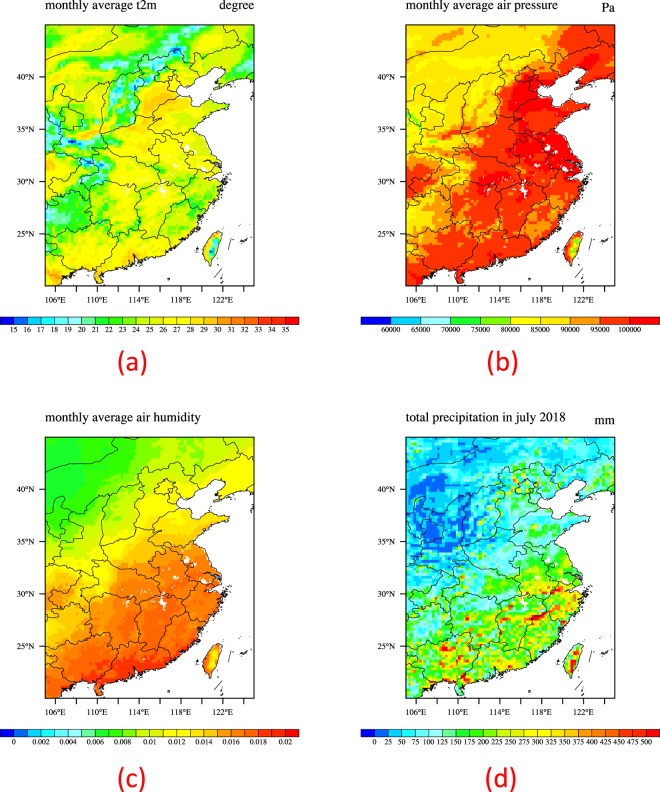
Monthly mean 2 m air temperature (**a**), monthly mean near surface air pressure (**b**), monthly mean 2 m air relative humidity (**c**) and total precipitation (**d**) of GLDAS in July 2018.

**Figure 3:**
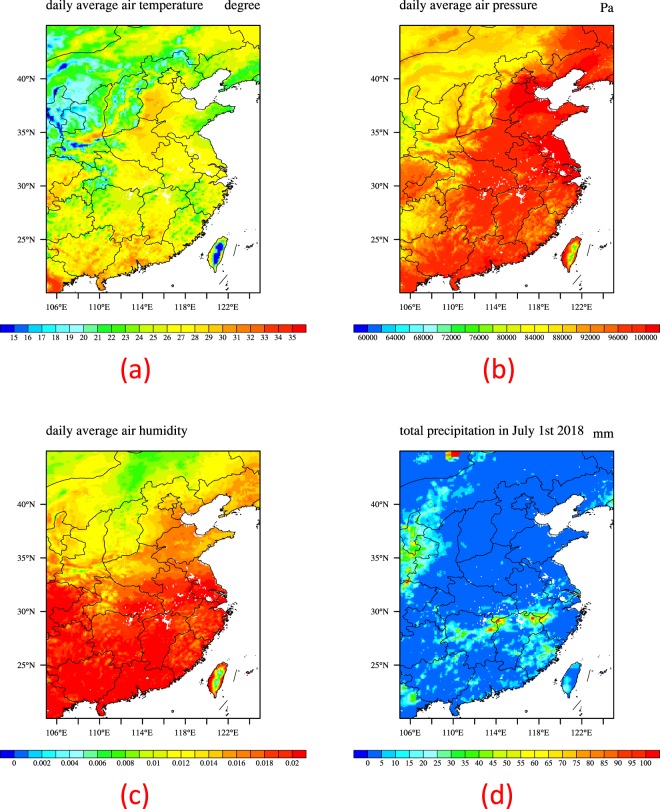
Daily mean 2 m air temperature (**a**), daily mean near surface air pressure (**b**), daily mean 2 m air relative humidity (**c**) and total precipitation (**d**) of CLDAS-v2.0 in July 1^st^ 2018.

The land use and land cover (LULC) data of eastern China are from MODIS in 2017; the spatial resolution is 0.05° (Fig. 1). For LSA and LSE assimilation, the atmospheric forcing data used to drive the land surface model are from the Global Land Data Assimilation System (GLDAS)^39^. The spatial and temporal resolution of GLDAS data are 0.25° and 3 hr, respectively. The GLDAS data were interpolated spatially and temporally to 0.05° and 1 hr by using a bilinear and cubic spline interpolation method, respectively. Figure 2 are the monthly mean 2 m air temperature, monthly mean near surface air pressure, monthly mean 2 m air relative humidity and total precipitation of GLDAS in July 2018, respectively. For LST assimilation, the atmospheric forcing data used to drive the land surface model are from the China Meteorological Administration (CMA) Land Data Assimilation System (CLDAS-v2.0)^40^. The spatial and temporal resolution of GLDAS data are 0.0625° and 1 hr, respectively. The CLDAS-v2.0 data were interpolated spatially to 0.05° by using a bilinear interpolation method. Figure 3 are the daily mean 2 m air temperature, daily mean near surface air pressure, daily mean 2 m air relative humidity and total precipitation of CLDAS-v2.0 in July 1^st^ 2018, respectively. For LST assimilation, the MODIS LSE data are used as the forcing variable.

#### Data used for assimilation

**Figure 4:**
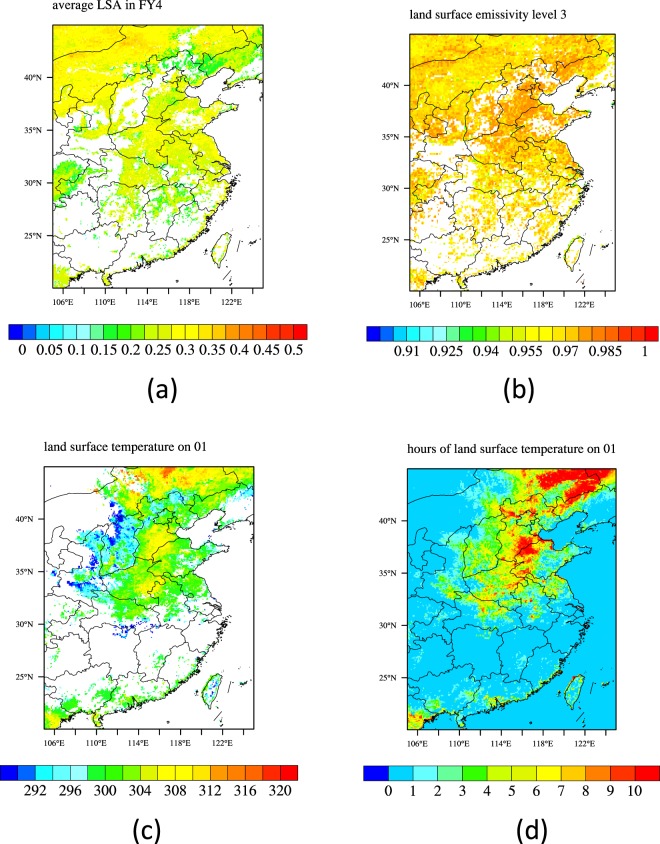
Data for Assimilation. (**a**) Fengyun-4A Monthly averages LSA in July 2018; (**b**) Fengyun-4A Monthly average LSE in July 2018; (**c**) Fengyun-4A average LST between 08:00 to 16:00 local time; (**d**) Times of the available Fengyun-4A LST data between 08:00 to 16:00 local time. (Generated by NCAR Command Language (NCL) Version 6.3.0, ref. ^44^)

The Fengyun-4A LSA, LSE and LST data are used for assimilation (Fig. 4). The time ranges of the LSA and LSE data are both a whole month of July 2018. The spatial and temporal resolutions of Fengyun-4A LSA are 3 km and 1d, respectively. The spatial and temporal resolutions of Fengyun-4A LSE are 12 km and 1d respectively. As the daily LSA and LSE don’t varied much in July 2018, the monthly average LSA and LSE were used to perform the assimilation. The monthly average LSA and LSE data were interpolated to 0.05° by using a bilinear interpolation method. The time range of the LST data is a whole day of 1^st^ July 2018. The spatial and temporal resolutions of Fengyun-4A LST are 4 km and 1 hr, respectively. The LST data were interpolated spatially to 0.05° by using a bilinear interpolation method.

#### Data used for validation

**Figure 5:**
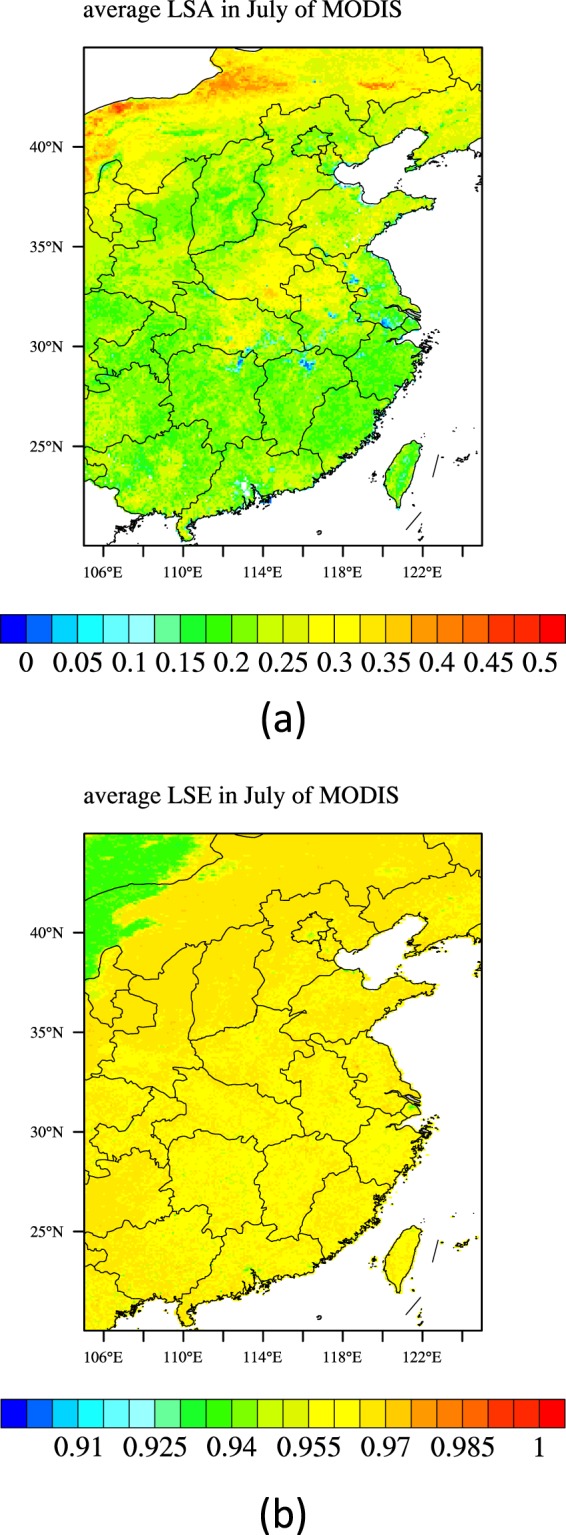
Data for validation. (**a**) MODIS Monthly averages near infra-red LSA in July 2018; (**b**) MODIS Monthly average LSE in July 2018. (Generated by NCAR Command Language (NCL) Version 6.3.0, ref. ^44^).

**Figure 6:**
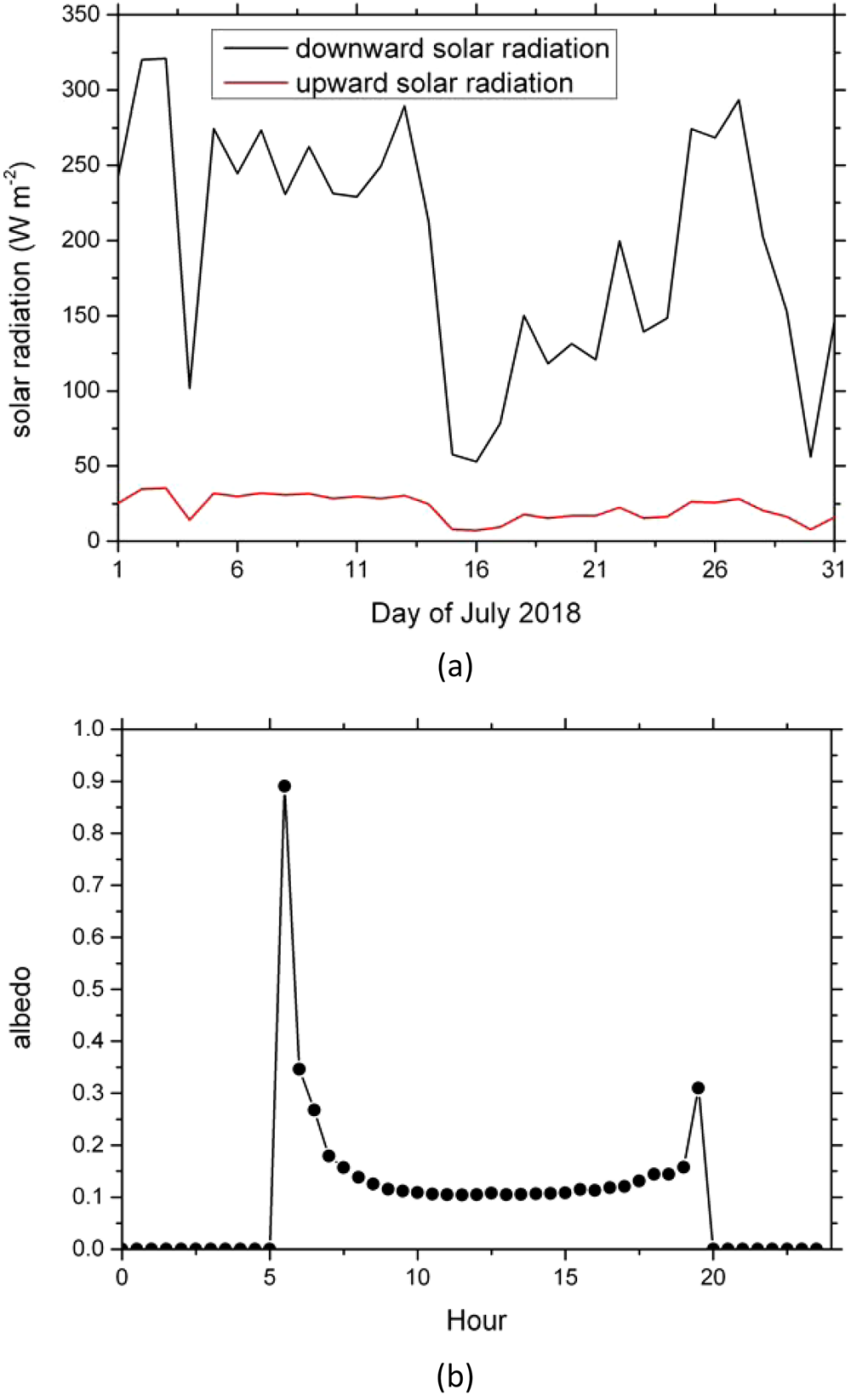
(**a**) Time series of the observed daily mean upward and downward shortwave radiation in IAP 325 m tower during July 2018. (**b**) Diurnal cycle of the albedo in IAP 325 m tower during July 2018.

The MODIS LSA and LSE data are used for validation (Fig. 5). The time ranges of the LSA and LSE data are both a whole month of July 2018. The IAP 325 m tower data were used for LSA comparison. The tower is located in downtown Beijing, the altitude of the foot of the tower is 49 m, the longitude and latitude are 116.3708E and 39.9744 N respectively. The radiation fluxes of the tower including the upward and downward shortwave and longwave radiation are measured using the radiometer at the 47-meter height. The time ranges of the observed upward and downward shortwave radiation are a whole month of July 2018 (Fig. 6(a)). The observed LSA (Fig. 6(b)) is calculated as follows:

1 $$\alpha =\frac{S\uparrow }{S\downarrow }$$

Where *α* is the surface albedo; $S\,\uparrow$ is the upward shortwave radiation (W m^−2^); $S\,\downarrow$ is the downward shortwave radiation (W m^−2^). The 5 cm and 10 cm soil temperature observation data in more than 100 sites in the research region were used for LST validation. The time ranges of the observed 5 cm and 10 cm soil temperature data are a whole day of 1^st^ July 2018. These observation data were downloaded from the National Meteorological Information Center. Table 1 lists the data used for the paper.

**Table 1:** Data used in this paper.

	Driving and Forcing	Assimilation	Validation
LSA Assimilation	GLDAS	Fengyun-4A LSA	MODIS LSA; IAP 325 m tower LSA
LSE Assimilation	GLDAS	Fengyun-4A LSE	MODIS LSE
LST Assimilation	CLDAS-v2.0	Fengyun-4A LST	Fengyun-4A LST; 5 cm and 10 cm observed soil temperature

## Methods

### Model

The IUM^35^ is used to perform the assimilation. The IUM was developed based on the Common Land Model (CoLM)^41^; it integrates the land surface models for urban and natural land surfaces. For the natural land surface, a whole layer soil evaporation parameterization scheme was developed to improve the simulation of soil evaporation especially in arid areas. For the urban land surface, the energy and water balance model were modified. As this paper only discusses the assimilation of the LSA, LSE and LST, only the equations which associated with the LSA, LSE and LST are listed in the assimilation method section.

In the IUM, the LST was calculated based on the surface energy balance equation. Expressing the conductive flux by Fourier’s law, the energy equation in ground surface becomes:

2 $$c{\Delta }z\frac{\partial {T}_{g}}{\partial t}=\frac{{t}_{k}}{{\Delta }z}({T}_{2}-{T}_{g})+{R}_{n,g}-{H}_{g}-L{E}_{g}$$

Where c is soil volumetric heat capacity (J m^−3^ k^−1^), *t*_*k*_ is the thermal conductivity (W m^−1^ k^−1^); *Δz* is the thickness of the surface soil layer (m); *T*_*g*_ is ground surface temperature (K); *T*_2_ is the second layer soil temperature (K); *R*_*n,g*_, *h*_*g*_ and *LE*_*g*_ are net radiation, sensible heat flux and latent heat flux (W m^−2^) respectively.

Foliage energy balance equations can be written as follows:

3 $${R}_{n,csun}-{H}_{csun}-L{E}_{csun}=0$$

4 $${R}_{n,csha}-{H}_{csha}-L{E}_{csha}=0$$

Where ${R}_{n,csun}$, ${R}_{n,csha}$ is net radiation absorbed by sunlit canopy and shaded canopy (W m^−2^) respectively; ${H}_{csun}$, ${H}_{csun}$ are sensible heat flux from sunlit canopy and shaded canopy (W m^−2^)) respectively; $L{E}_{csun}$, $L{E}_{csun}$ are latent heat flux from sunlit canopy and shaded canopy (W m^−2^) respectively.

In the IUM, the LSA is defined as follows:

5 $$\alpha =(1-{f}_{\mathrm{cov}er}){\alpha }_{soil}+{f}_{\mathrm{cov}er}{\alpha }_{veg}$$

Where *α* is the LSA, ${f}_{\mathrm{cov}er}$ is the fractional vegetation cover, ${\alpha }_{soil}$ and ${\alpha }_{veg}$ are the LSA for bare soil and vegetation, respectively. For bare soil, the LSA is associated with the soil wetness and color. For vegetation, the LSA is calculated using a two stream approximation scheme.

In the IUM, the LSE was associated with the LULC. It is set as 0.97 for snow, glaciers and water surface; 0.96 for soil and wetland; and 1 for vegetated surfaces.

### Organization of the model and the data assimilation method

#### Model initialization

As the initial states such as the soil temperature, soil moisture and snow depth etc. are not available in the study region. A spin-up was run to obtain relatively accurate initial values for the initial states. The spin-up time period is from 0:00 1^st^ January to 0:00 1^st^ July 2018. During the spin-up period, the atmospheric forcing data used to drive the land surface model are from the GLDAS. The GLDAS data were interpolated spatially and temporally to 0.05° and 1 hr by using a bilinear and cubic spline interpolation method, respectively. The initial values of the IUM including the soil temperature and soil moisture profile, snow depth are all from the GLDAS.

#### LSA and LSE assimilation

Both LSA and LSE are associated with the radiation balance equation. The distribution of the radiation energy is changed by the LSA and LSE through the adjustment of the net radiation, which is the key state in surface radiation balances (Eq. 6).

6 $${R}_{n,g}=(1-\alpha )S\downarrow +(1-{\varepsilon }_{g})L\downarrow -{\varepsilon }_{g}\sigma {T}_{g}^{4}$$

Where $S\,\downarrow$ is the downward shortwave solar radiation (W m^−2^), ${\varepsilon }_{g}$ is the emissivity of the ground surface, $L\,\downarrow$ is the downward longwave radiation (W m^−2^), $\sigma$ is the Stefan-Boltzmann constant (W m^−2^ K^−4^).

The direct insertion method was used to perform the LSA and LSE assimilation. A whole month of July 2018 was used as the assimilation window. For the areas where the Fengyun-4A LSA and LSE data were available, the calculation module of the LSA and LSE in the IUM were replaced directly by the monthly average LSA and LSE data from Fengyun-4A, respectively. The MODIS and the IAP 325 m tower LSA data are used for validation. The MODIS LSE data are used for validation.

#### LST assimilation

As the downward longwave radiation data is not included in CLDAS-v2.0, an estimation method^42^ was used.

7 $${R}_{c}=[1-0.35\ast \exp (-\,10\ast \frac{e}{T})]\sigma {T}^{4}$$

8 $$R={R}_{c}(1+0.035\ast clou{d}^{2})$$

Where *R* is the downward longwave radiation (W m^−2^); *e* is the air vapor pressure (hPa); *T* is the near surface air temperature (K); ${R}_{c}$ is the downward longwave radiation in clear sky condition (W m^−2^); *cloud* is the fractional cloud cover, which is calculated as follows.

9 $$cloud=\frac{1160.\ast \cos (\theta )-S\downarrow }{963.\ast \cos (\theta )}\,\,\,0\le cloud\le 1$$

Where *θ* is the solar zenith angle.

As the parallel algorithms were not used in the LST assimilation, a whole day of 1^st^ July 2018 was used as the assimilation window. The time step of the assimilation is 1 hour. A variational approach is used to assimilate the LST into the IUM, and the surface energy balance equation is employed as the adjoint physical constraint. The variational assimilation approach assimilates the LST through the adjustment of the EFs. The corresponding cost function *J* is:

10 $$\begin{array}{rcl}J({T}_{g},{T}_{lsun},{T}_{lsha},{\gamma }_{ss},{\gamma }_{sv},{\gamma }_{v},{\lambda }_{1},{\lambda }_{2},{\lambda }_{3}) & = & \frac{{C}_{1}}{2}{({T}_{R}({t}_{1})-{T}_{obs}({t}_{1}))}^{2}+\frac{{C}_{T}}{2}{{\int }_{{t}_{0}}^{{t}_{1}}({T}_{R}-{T}_{obs})}^{2}dt\\  &  & +\,\frac{{C}_{{\gamma }_{ss}}}{2}{{\int }_{{t}_{0}}^{{t}_{1}}({\gamma }_{ss}-{\gamma }_{ss}^{^{\prime} })}^{2}dt+\frac{{C}_{{\gamma }_{sv}}}{2}{{\int }_{{t}_{0}}^{{t}_{1}}({\gamma }_{sv}-{\gamma }_{sv}^{^{\prime} })}^{2}dt\\  &  & +\,\frac{{C}_{{\gamma }_{v}}}{2}{{\int }_{{t}_{0}}^{{t}_{1}}({\gamma }_{v}-{\gamma }_{v}^{^{\prime} })}^{2}dt\\  &  & +\,{\int }_{{t}_{0}}^{{t}_{1}}{\lambda }_{1}[c\varDelta z\frac{\partial {T}_{g}}{\partial t}-\frac{{t}_{k}}{\Delta z}({T}_{2}-{T}_{g})-{R}_{n,g}+{H}_{g}\\  &  & +\,L{E}_{g}]dt+{\int }_{{t}_{0}}^{{t}_{1}}{\lambda }_{2}({H}_{csun}+L{E}_{csun}-{R}_{n,csun})dt\\  &  & +\,{\int }_{{t}_{0}}^{{t}_{1}}{\lambda }_{3}({H}_{csha}+L{E}_{csha}-{R}_{n,csha})dt\end{array}$$

The parameters *T*_*obs*_ and *T*_*R*_ are the LST measured by observation and simulated by IUM respectively. The parameters *γ*_*SS*_, *γ*_*sv*_ and *γ*_*v*_ are, respectively, EF of bare soil, ground surface under the canopy and canopy. The sixth, seventh and eighth terms on the right hand of Eq. (10) are adjoint physical constraints of ground surface, sunlit canopy and shaded canopy respectively. The parameters *λ*_1_, *λ*_2_ and *λ*_3_ are Lagrange multipliers. *C*_1_, *C*_*T*_, ${C}_{{\gamma }_{ss}}$, ${C}_{{\gamma }_{sv}}$ and ${C}_{{\gamma }_{v}}$ in the cost function are weight of the different terms, often defined as the inverse of the covariance matrix. For the simplicity of the algorithm, these weights are assumed to be constants in this paper. As the EFs are not varied much from 08:00 to 16:00 local time, only the observed LST data within this time period (Fig. 4(c)) were assimilated into the IUM. In the northeast areas, the times of the available LST data were relative large (Fig. 4(d)). The Fengyun-4A LST data and the observed 5 cm and 10 cm soil temperature data are used for validation.

## Results and Discussion

**Figure 7:**
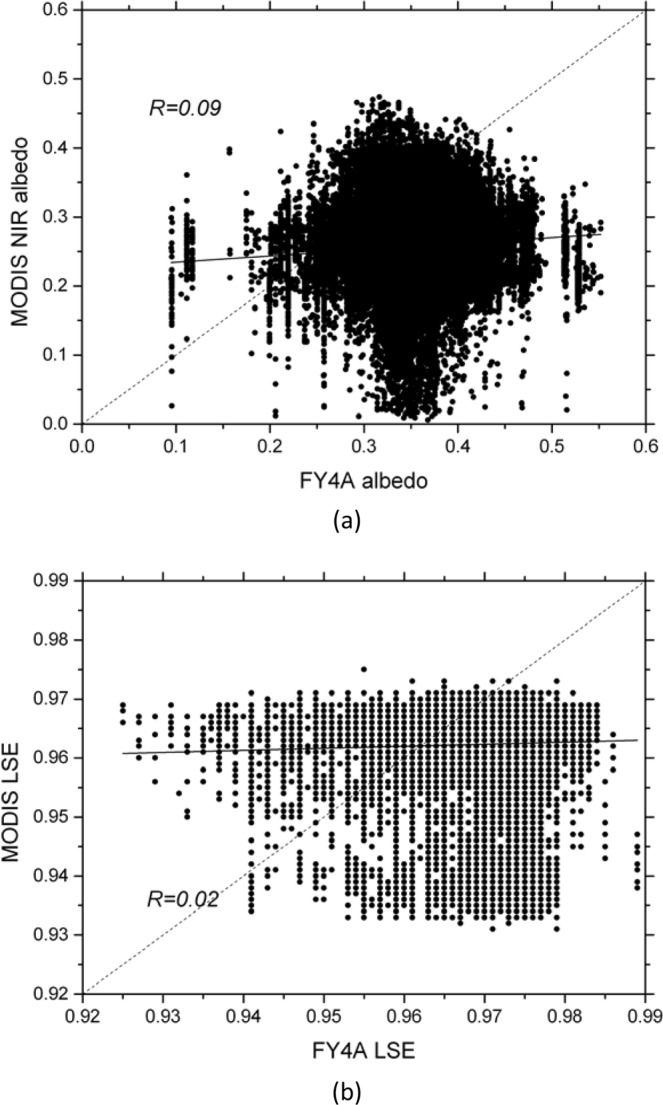
Scattered plots of the LSA (**a**) and LSE (**b**) between the Fengyun-4A and MODIS (Generated by Origin 9.0 http://originlab.com/).

First, in order to validate the accuracy of the LSA and LSE products from Fengyun-4A, we compared them with the observed LSA in 325 m tower site and the MODIS LSE and near-infrared LSA. We used the LSE in three wave bands, i.e. 29, 31 and 32 to estimate the total LSE^43^. For most of the areas in the research region, the MODIS near-infrared LSA is smaller than the Fengyun-4A (Fig. 7(a)). The spatially average bias is 0.092; the mean absolute error (MAE) is 0.097; the root mean square error (RMSE) is 0.116. There are nearly no correlation between them, the correlation coefficient is 0.09; the Kling-Gupta Efficiency (KGE) score is −0.018. As the near-infrared LSA is bigger than the visible LSA, the total MODIS LSA is much smaller than the Fengyun-4A. From Eq. 1, the monthly average LSA for 325 m tower site is 0.114; while the Fengyun-4A LSA is 0.272, the MODIS near-infrared LSA is 0.154. If we suppose the visible LSA is a half of the near-infrared LSA and the percentage of the visible and near-infrared solar radiation are equal, the total MODIS LSA is 0.115. It could be concluded the MODIS LSA is superior to the Fengyun-4A apparently. As for the LSE, the difference between MODIS and Fengyun-4A is relatively small (Fig. 7(b)). The spatially average bias is 0.0063; the mean absolute error (MAE) is 0.0093; the root mean square error (RMSE) is 0.0128. There are nearly no correlation between them, the correlation coefficient is 0.02; the Kling-Gupta Efficiency (KGE) score is 0.0101.

**Figure 8:**
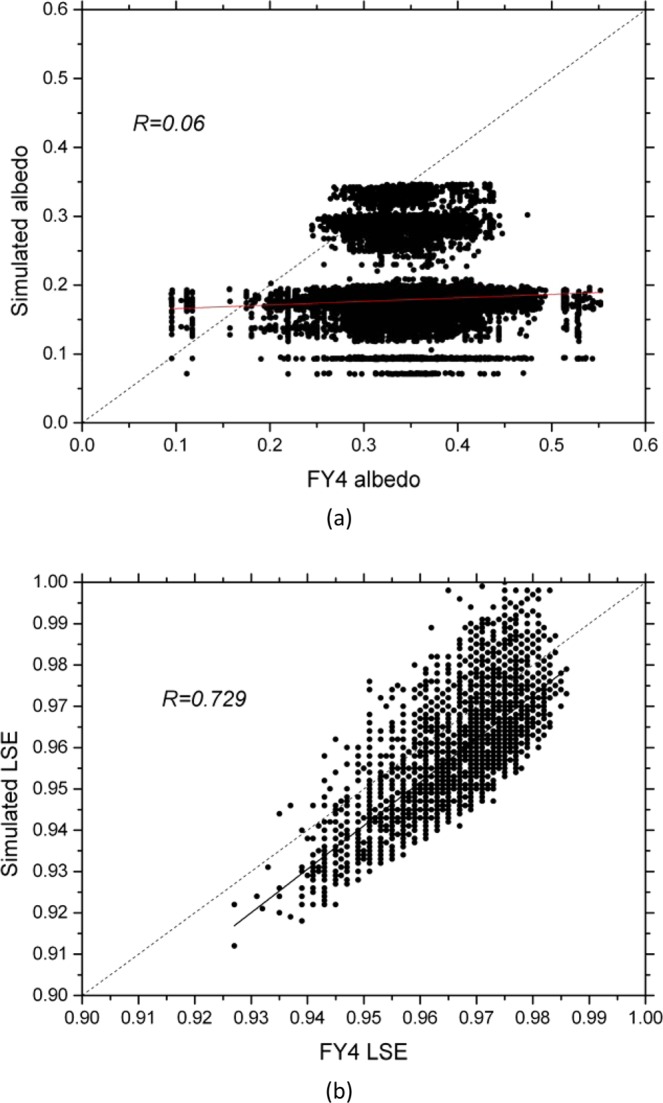
Scattered plots of the LSA (**a**) and LSE (**b**) between the Fengyun-4A and the simulation. (Generated by Origin 9.0 http://originlab.com/).

**Figure 9:**
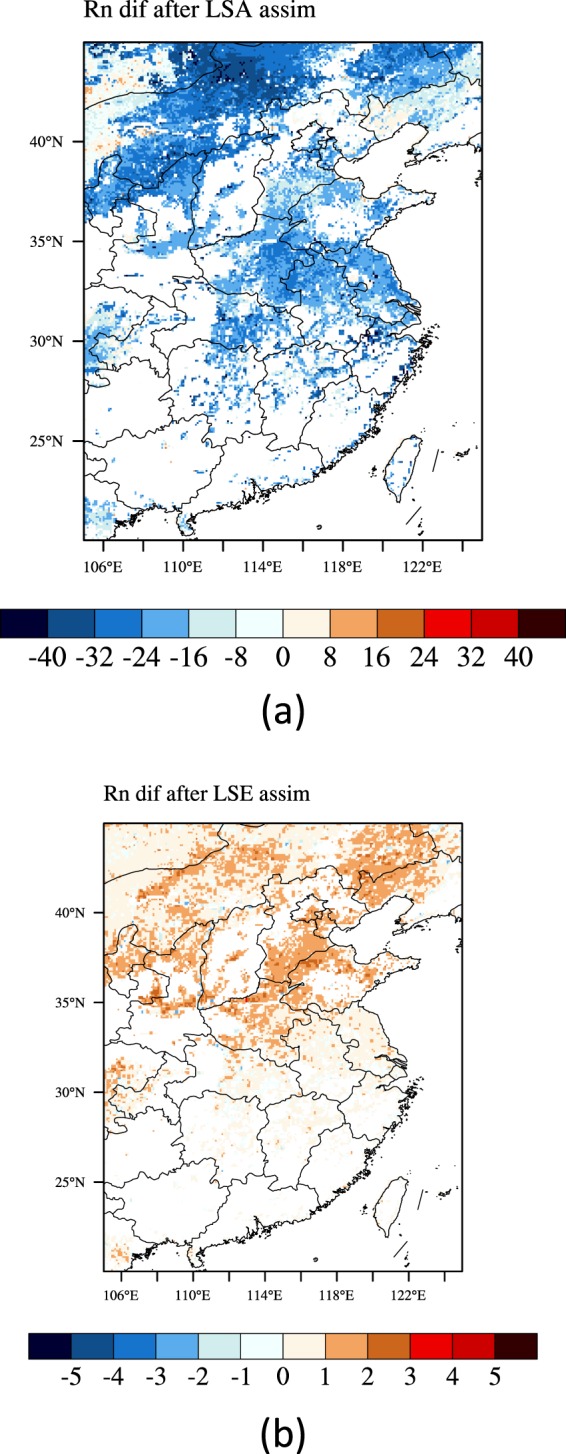
Net radiation differences between the simulations before and after LSA (**a**) and LSE (**b**) assimilation (after assimilation subtract before assimilation) (Generated by NCAR Command Language (NCL) Version 6.3.0, ref. ^44^).

**Figure 10:**
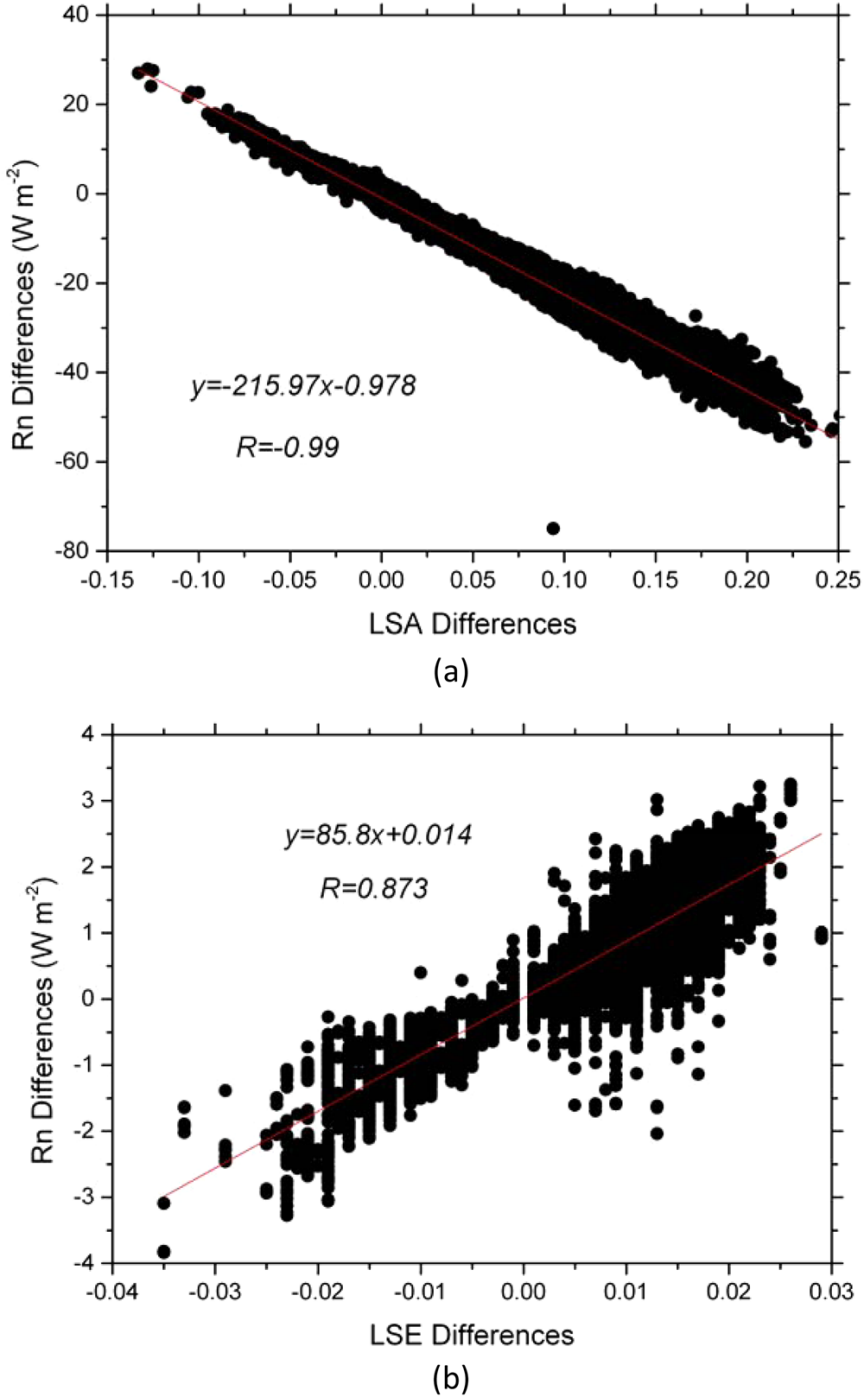
Scattered plots of the LSA (**a**) and LSE differences (**b**) compared with the net radiation differences before and after assimilation (after assimilation subtract before assimilation) (Generated by Origin 9.0 http://originlab.com/).

Then we assimilated the Fengyun-4A LSA and LSE into the IUM separately and compared the simulation results before and after assimilation. As the net radiation is the key state in surface radiation and energy balances, we compared the net radiation simulation results before and after the LSA and LSE assimilation separately. In most regions, the Fengyun-4A LSA (Fig. 8(a)) and LSE (Fig. 8(b)) are larger than those of the simulations. The spatially average bias of the LSA is 0.17; the mean absolute error (MAE) is 0.17; the root mean square error (RMSE) is 0.183. There are nearly no correlation between them, the correlation coefficient is 0.05; the Kling-Gupta Efficiency (KGE) score is −0.412. As for the LSE, the spatially average bias is 0.008; the mean absolute error (MAE) is 0.01; the root mean square error (RMSE) is 0.011. They have significantly positive correlation, the correlation coefficient is 0.729; the Kling-Gupta Efficiency (KGE) score is 0.586. As the result, after the LSA assimilation, in most regions the simulated net radiation was decreased (Fig. 9(a)). The correlation coefficient between the LSA differences and the net radiation differences is 0.99 (Fig. 10(a)). After the LSE assimilation, in most regions the simulated net radiation was increased (Fig. 9(b)). The correlation coefficient between the LSE differences and the net radiation differences is 0.873 (Fig. 10(b)). The LSE has dual influences on the net radiation (Eq. 6). Firstly, the increase of the LSE increases the upward longwave radiation and then decreases the net radiation. Secondly, the increase of the LSE decreases the reflection of the downward longwave radiation and then increases the net radiation.

**Figure 11:**
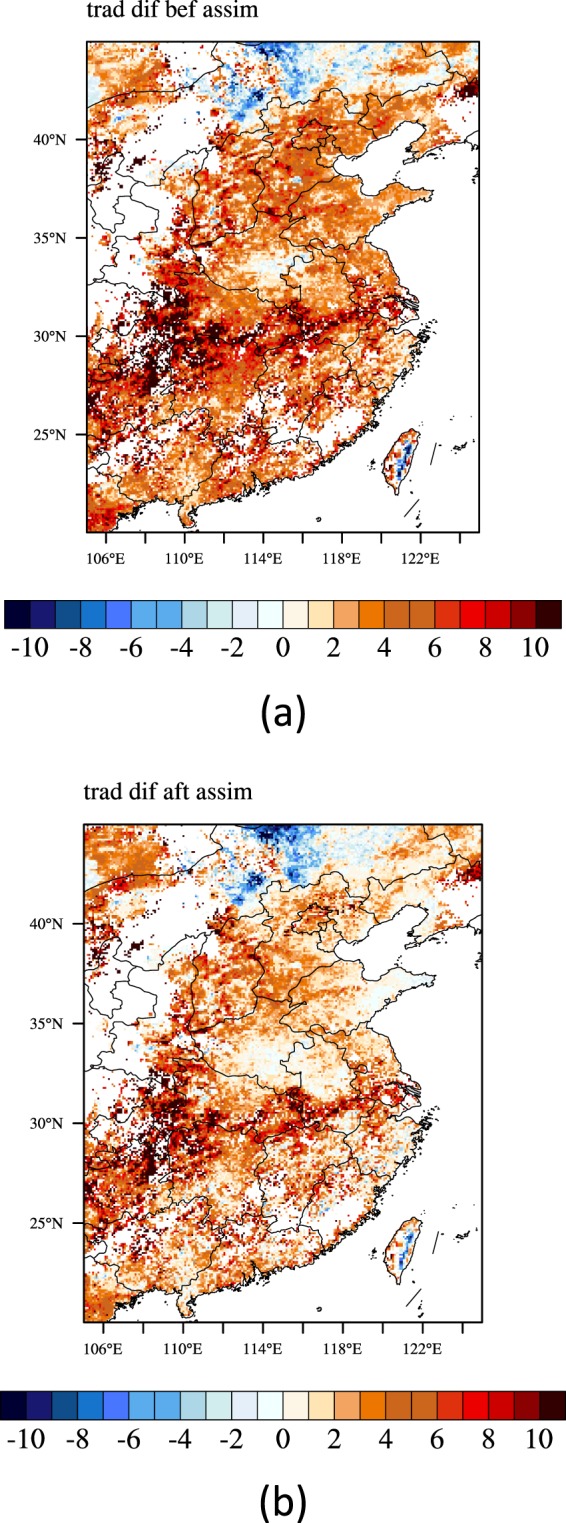
Daily average LST biases between the simulations before (**a**) and after (**b**) 546 assimilation and the Fengyun-4A (simulation subtract Fengyun-4A) (Generated by 547 NCAR Command Language (NCL) Version 6.3.0, ref. ^44^).

**Figure 12:**
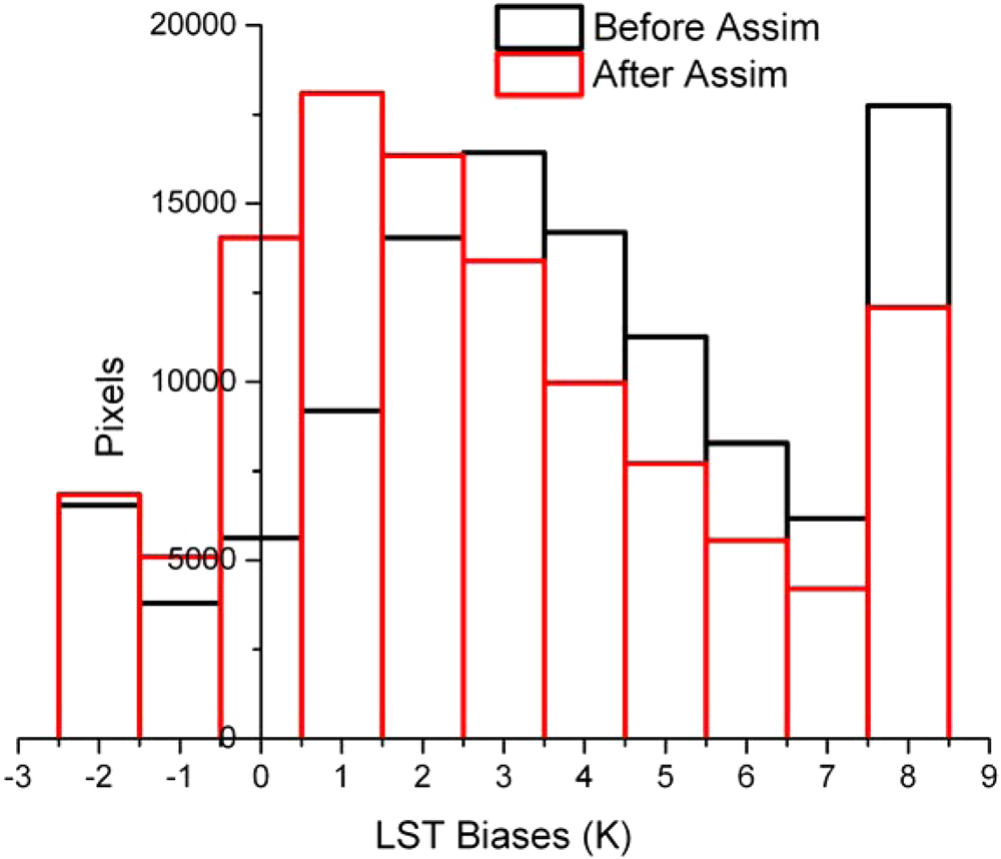
Histogram of the biases in the study regions where Fengyun-4A LST data were available between the simulations before and after assimilation and the Fengyun-4A (simulation subtract Fengyun-4A) (Generated by Origin 9.0 http://originlab.com/).

**Figure 13:**
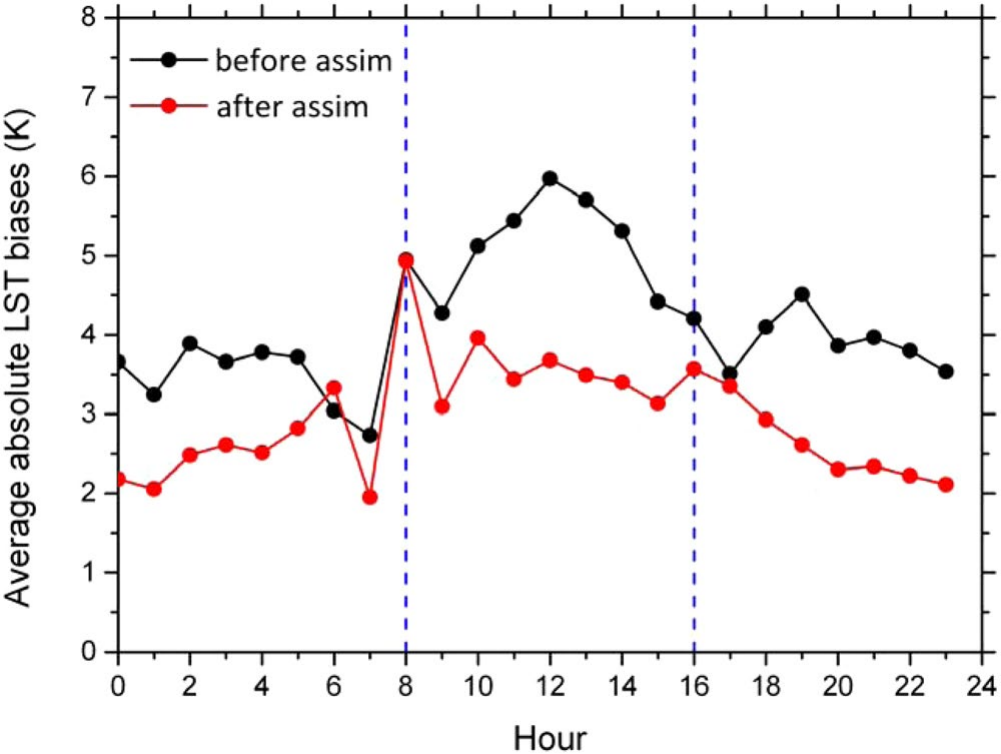
Time series of the resulting average absolute LST biases in the study regions where Fengyun-4A LST data were available between the simulations before and after assimilation and the Fengyun-4A (simulation subtracts Fengyun-4A). (Generated by Origin 9.0 http://originlab.com/).

Then we assimilated the Fengyun-4A LST into the IUM and compared the simulation results before and after assimilation with the Fengyun-4A. Figure 11 is the daily average LST biases between the simulations before and after assimilation and the Fengyun-4A (simulation subtracts Fengyun-4A). In the north part of the research area where more times of the Fengyun-4A LST data were available, after assimilation, the biases of the LST were decreased apparently. In order to compare the simulations before and after assimilation quantitatively, histograms of the LST biases (simulation subtracts Fengyun-4A) are plotted in Fig. 12. After assimilation, the numbers of the relatively big biases pixels (such as more than 7 K) were decreased; while the numbers of the relatively small biases pixels were increased (such as from −1 to 2 K). Figure 13 is the time series of the resulting average absolute LST biases in the study regions where Fengyun-4A LST data were available between the simulations before and after assimilation and the Fengyun-4A (simulation subtracts Fengyun-4A). After assimilation, the average absolute LST biases were decreased for most of the hours except 6:00 am because the EFs would fluctuate especially at dawn and dusk. For most of the hours expect at dawn and dusk, the biases were decreased about 20 to 40 percent. As a whole, the average absolute LST bias was decreased about 29.3 percent.

**Figure 14:**
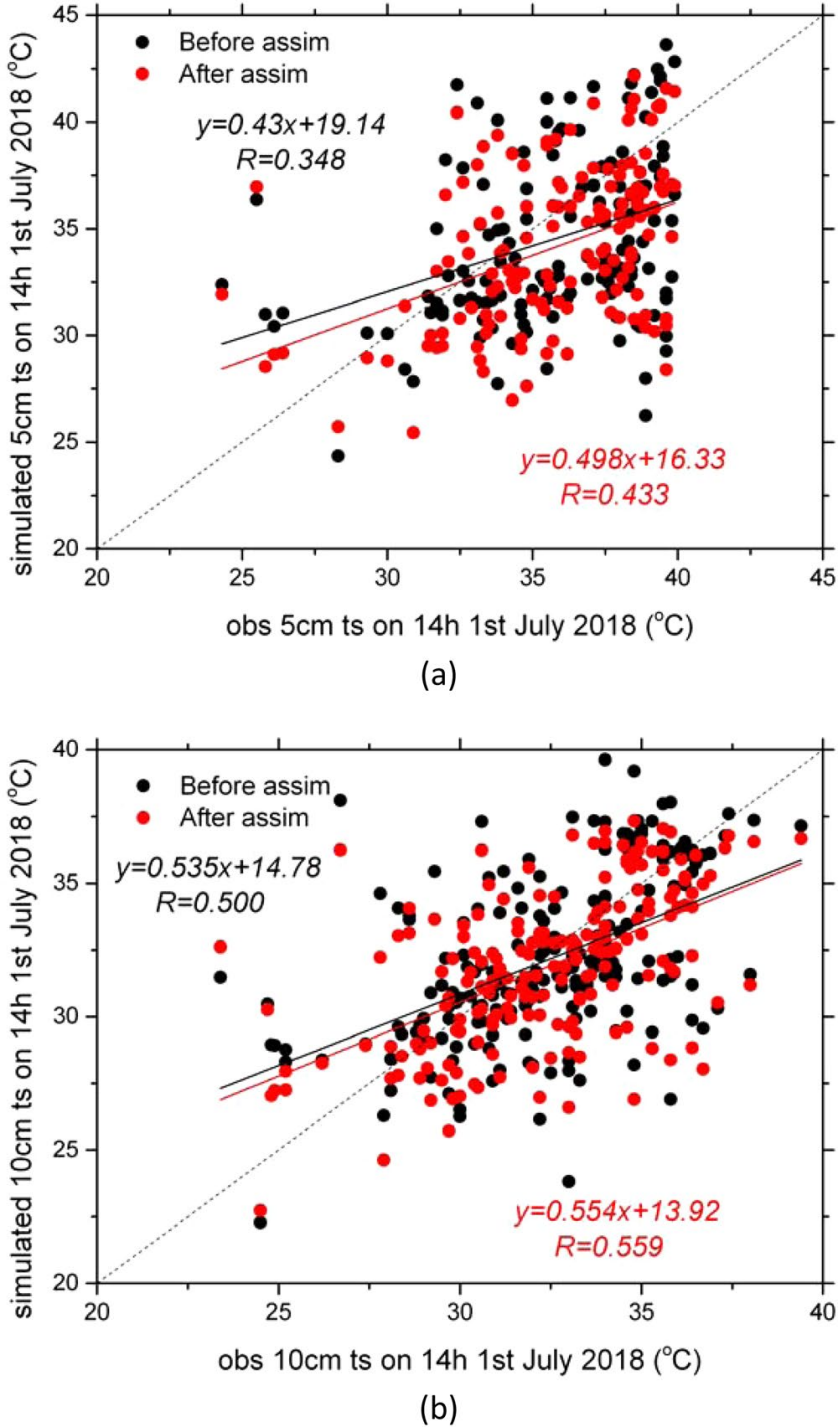
Scattered plots of the daily average observed 5 cm (**a**) and 10 cm (**b**) soil temperature compared with the simulations before and after LST assimilation (Generated by Origin 9.0 http://originlab.com/).

**Figure 15:**
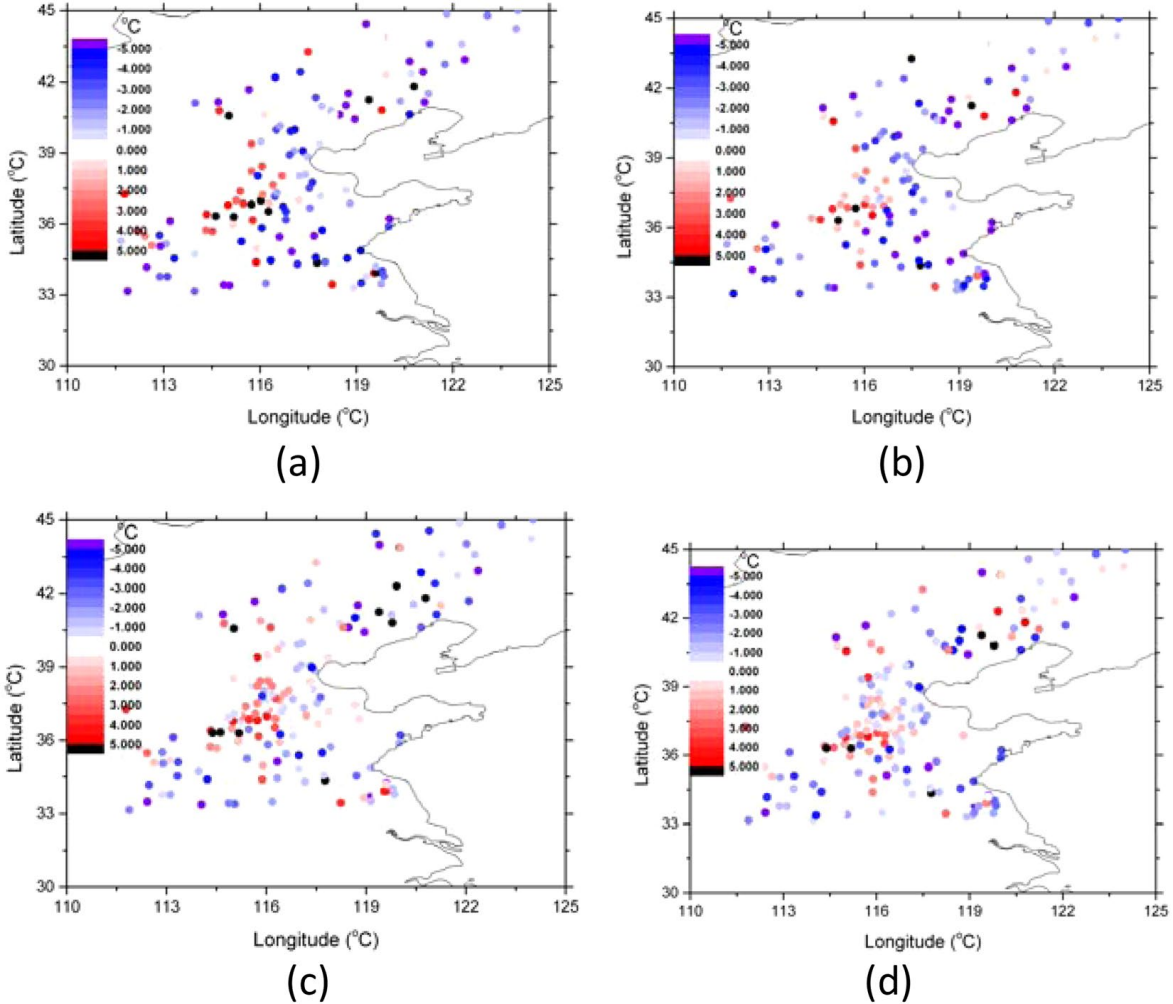
Biases between the daily average simulated and observed soil temperature (simulation subtract observation). (**a**) 5 cm before assimilation; (**b**) 5 cm after assimilation; (**c**) 10 cm before assimilation; (**d**) 10 cm after assimilation (Generated by Origin 9.0 http://originlab.com/).

In order to validate the effect of the LST assimilation on the soil temperature, the 5 cm and 10 cm soil temperature observation data in more than 100 sites in the north part of the research area (30N–45N) where Fengyun-4A LST data were available were used for comparison (Figs. 14 and 15, Table 2). The simulated soil temperature data were interpolated linearly to 5 cm and 10 cm. After assimilation, the correlation coefficient between the observed and simulated 5 cm soil temperature was increased from 0.348 to 0.433; the correlation coefficient between the observed and simulated 10 cm soil temperature was increased from 0.500 to 0.559. After assimilation, the daily average biases of the 5 cm and 10 cm soil temperature were decreased from 3.54 to 3.29 and from 2.31 to 2.10 respectively.

**Table 2:** Mean biases between the simulated and observed soil temperature (simulations subtract observations).

	5 cm before (K)	5 cm after (K)	10 cm before (K)	10 cm after (K)
Mean biases	3.54	3.29	2.31	2.10

## Conclusions

In this paper, the LSA, LSE and LST data from Fengyun-4A were assimilated into the IUM. The Fengyun-4A LSA and LSE data were compared with the MODIS retrieved data and the both the LSA data were validated using the IAP 325 m tower observation data.

The results indicate the MODIS LSA is much smaller than the Fengyun-4A and is superior to the Fengyun-4A for the IAP 325 m tower site. The MODIS LSE is smaller than the Fengyun-4A in barren LULC and the differences is relatively small for other LULCs. In most regions of the research area, the Fengyun-4A LSA and LSE are larger than those of the simulations. As the result, after the LSA assimilation, in most regions the simulated net radiation was decreased. After the LSE assimilation, in most regions the simulated net radiation was increased. In the north part of the research area where more times of the Fengyun-4A LST data were available, after assimilation, the biases of the LST were decreased apparently. After assimilation, the correlation coefficients between the observed and simulated 5 cm and 10 cm soil temperature were increased; the biases of the daily average 5 cm and 10 cm soil temperature were decreased.

In the near future, the LSA, LSE and LST data from Fengyun-4A will be validated using multi-sites observations. The inverse error covariances of the cost function should be reparemeterized to optimize the assimilation results in regional scale. The dimensionless bulk heat transfer coefficient under neutral conditions should be combined with the EFs as the tuned factors.
